# Reducing HIV-related stigma among young people attending school in Northern Uganda: study protocol for a participatory arts-based population health intervention and stepped-wedge cluster-randomized trial

**DOI:** 10.1186/s13063-022-06643-9

**Published:** 2022-12-23

**Authors:** Joshua B. Mendelsohn, Bonnie Fournier, Stéphanie Caron-Roy, Geoffrey Maina, Gillian Strudwick, Santo Ojok, Hyun June Lim, Marcos Sanches, Carmen H. Logie, Susan Sommerfeldt, Candace Nykiforuk, Jean Harrowing, Francis Akena Adyanga, Jussy Okello Hakiigaba, Olenka Bilash

**Affiliations:** 1 College of Health Professions, Pace University, 163 William Street, New York, NY 10038 USA; 2 School of Nursing, Thompson Rivers University, 805 TRU Way, Kamloops, BC V2C 0C8 Canada; 3 College of Nursing, University of Saskatchewan, Health Science Building - 1A10, Box 6, 107 Wiggins Road, Saskatoon, Saskatchewan S7N 5E5 Canada; 4 Centre for Addiction and Mental Health, 1001 Queen Street West, Toronto, Ontario M6J 1H1 Canada; 5 Institute of Health Policy, Management and Evaluation, University of Toronto, 4th Floor, 155 College Street, Toronto, Ontario M5T 3M6 Canada; 6 Tochi Youth Resource Centre, PO Box 416, Gulu, Uganda; 7 Department of Community Health & Epidemiology, University of Saskatchewan, Health Science Building, 107 Wiggins Road, Saskatoon, Saskatchewan S7N 5E5 Canada; 8 Krembil Centre for Neuroinformatics, Centre for Addiction and Mental Health, 250 College Street, Toronto, Ontario M5T 1R8 Canada; 9 Factor-Inwentash Faculty of Social Work, University of Toronto, 246 Bloor Street W, Toronto, Ontario M5S 1V4 Canada; 10 Women’s College Hospital, 76 Grenville Ave, Toronto, ON M5S 1B2 Canada; 11 Faculty of Nursing, University of Alberta, 11405 - 87 Ave, Edmonton, Alberta T6G 1C9 Canada; 12 School of Public Health, University of Alberta, 11405 – 87 Ave, Edmonton, Alberta T6G 1C9 Canada; 13 Faculty of Health Sciences, University of Lethbridge, 4401 University Drive, Lethbridge, Alberta T1K 3M4 Canada; 14 Faculty of Education, Kabale University, Plot 364 Block 3 Kikungiri Hill, Kabale Municipality, Uganda; 15 Faculty of Education, University of Alberta, 249 Education Centre – South, 11210 - 87 Ave NW, Edmonton, Alberta T6G 2G5 Canada

**Keywords:** HIV, Stigma, Children, Adolescents, Young people, Uganda, Stepped-wedge cluster-randomized trial (SW-CRT), Protocol, Schools, Arts-based intervention

## Abstract

**Background:**

HIV-related stigma negatively impacts HIV prevention, treatment, and care, particularly among children and adolescents in sub-Saharan Africa. Interventions that are culturally grounded and relevant for addressing root causes may reduce the stigma experienced by HIV-positive and HIV-affected young people. This study, to be conducted in a post-conflict, rural setting in Omoro District, Uganda, will develop and evaluate a transformative  arts-based HIV-related stigma intervention rooted in local cultural knowledge to reduce stigma and improve HIV prevention and care for young people living with HIV. The intervention will be delivered to young people attending school by community Elders, with the support of teachers, through the transfer of local cultural knowledge and practices with the aim of re-establishing the important cultural and social role of Elders within a community that has suffered the loss of intergenerational transfer of cultural knowledge throughout a 25-year civil war.

**Methods:**

A formative research phase consisting of interviews with students, teachers, and Elders will inform the intervention and provide data for study objectives. Workshops will be delivered to Elders and teachers in participating schools to build capacity for arts-based, educational workshops to be conducted with students in the classroom. The intervention will be evaluated using a stepped-wedge cluster-randomized trial. Government-funded schools in Omoro District will be randomized into three blocks, each comprised of two primary and two secondary schools (*n*=1800 students). Schools will be randomly assigned to a crossover sequence from control to intervention condition in 8-week intervals. A process evaluation will be implemented throughout the study to evaluate pathways between intervention development, implementation, and effects.

**Discussion:**

This study will generate comprehensive, in-depth participatory research and evaluation data to inform an effective and sustainable protocol for implementing arts-based HIV stigma interventions for young people in school settings. Findings will have widespread implications in post-conflict settings for HIV prevention, treatment, and care.

**Trial registration:**

ClinicalTrials.gov NCT04946071. Registered on 30 June 2021.

**Supplementary Information:**

The online version contains supplementary material available at 10.1186/s13063-022-06643-9.

## Background

Children and adolescents growing up with HIV infection or those living with parents or caregivers who live with HIV or AIDS experience various forms of stigma. Stigma refers to attitudes and beliefs that lead people to reject, avoid, or fear those they perceive as different and can lead to mistreatment and reduced access to power and opportunities [1]. HIV-related stigma continues to negatively impact HIV prevention, treatment, and care. For instance, HIV-related stigma has been associated with reduced uptake of voluntary counseling and testing [2], reduced likelihood of HIV status disclosure [3], and poses a significant barrier to antiretroviral therapy (ART) medication adherence, contributing to high rates of HIV mortality, especially in sub-Saharan Africa [4, 5]. Additionally, HIV stigma contributes to mental health issues in children and adolescents, such as anxiety and depression [6]. It may lead to school drop-out due to bullying, discrimination by peers and teachers and suicidal ideation [7]. As a result, young people face the dual burden of physical illness from living with the HIV virus and psychosocial distress resulting from stigma and discrimination.

Despite the importance of addressing HIV-related stigma and discrimination among young people, studies supporting interventions that directly address stigma among this population are limited [8, 9]. A recent systematic review found that, of the few stigma reduction interventions targeting HIV-positive adolescents in sub-Saharan Africa, intervention duration was between one-half of one day and one week, with longer duration interventions showing stronger effects [10]. The most common interventions provided information and skill-building sessions for HIV-negative individuals [11]. The findings of this review suggested that additional stigma reduction interventions beyond information and skill-building that address a wider variety of stigmas among young people over a longer duration. In a separate review of 48 stigma reduction interventions, only three aimed to reduce stigma among youth living with HIV in sub-Saharan Africa. Most studies were conducted with adults in Asia and the Pacific Region [11]. None targeted HIV-positive children in Northern Uganda. Arts-based intervention studies with young people that are rooted in participants’ ability to control the production and dissemination of knowledge have found reductions in internalized stigma through feelings of empowerment [12–15]. Interventions to reduce stigma need to be culturally grounded and relevant, if they are to change social and cultural norms that reproduce stigma [16]. In general, there is a dearth of evidence-based interventions to address HIV stigma in low- and middle-income countries like Uganda [17], and fewer studies still that have investigated arts-based methods among young people [18]. Given the limited evidence available in support of effective culturally grounded stigma reduction among young people and the push to end the AIDS epidemic by 2030 (Sustainable Development Goals Target 3.3), the identification of effective HIV stigma reduction interventions among this population could have widespread global implications for HIV prevention and care.

The present study will take place in Northern Uganda and will aim to generate much needed evidence for HIV stigma intervention research and practice by developing and evaluating a transformative arts-based intervention involving community members across all stages of the research process [19]. The intervention will be delivered to young people attending school by Elders with the support of teachers through the transfer of local cultural knowledge and practices. Findings will provide insights regarding the use of local cultural knowledge for informing arts-based interventions designed to reduce HIV stigma while improving HIV knowledge, attitudes, and practices among young people. This arts-based intervention will attempt to re-establish the important cultural and social role of Elders within a community that has suffered the loss of intergenerational transfer of cultural knowledge as a result of a 25-year long civil war.

### Objectives

The aim of this study is to design, implement and evaluate an arts-based intervention based on local cultural knowledge to reduce stigma and improve HIV outcomes among young people living with HIV or affected by HIV, aged ≥10 years old. The study is designed to achieve the following objectives:

1. Assess the magnitude of change in measures of stigma (i.e., enacted, internal, anticipated, courtesy, and perceived), HIV knowledge, and fear of transmission and disease among young people who are HIV-positive and HIV-affected and who receive the arts-based intervention;
2. Assess the intervention effect on linkage to care, initiation of ART, adherence to ART, and viral suppression among young people living with HIV;
3. Evaluate how and under what circumstances the intervention had an effect.

## Methods

### Study design

**Fig. 1:**
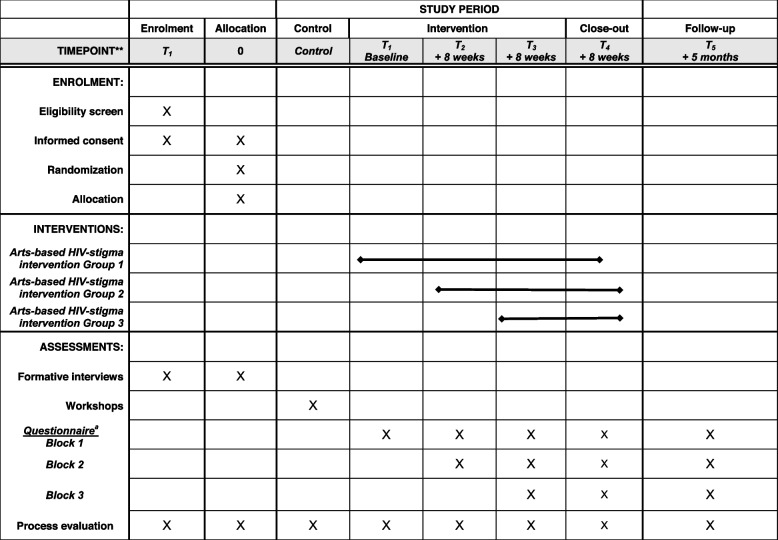
SPIRIT flow diagram

This trial protocol describes a participatory arts-based intervention to be implemented in schools and evaluated using a stepped-wedge cluster randomized trial. This protocol is reported in compliance with the Standard Protocol Items: Recommendations for Intervention Trials (SPIRIT) guidelines (see Fig. 1 and Additional file 1). A formative phase based on qualitative interviews will provide data to inform intervention content and delivery. The intervention will be developed using a series of community-based participatory research (CBPR) meetings with a Community Advisory Committee (CAC) and a group of Elders. A process evaluation occurring throughout the study will evaluate linkages between development, implementation, and intervention effects. The study was designed in partnership with the District Health Officer, the District Education Officer, the AIDS Support Organization (TASO), and the Waroco Kwo Elders Association in Omoro District.

**Fig. 2:**
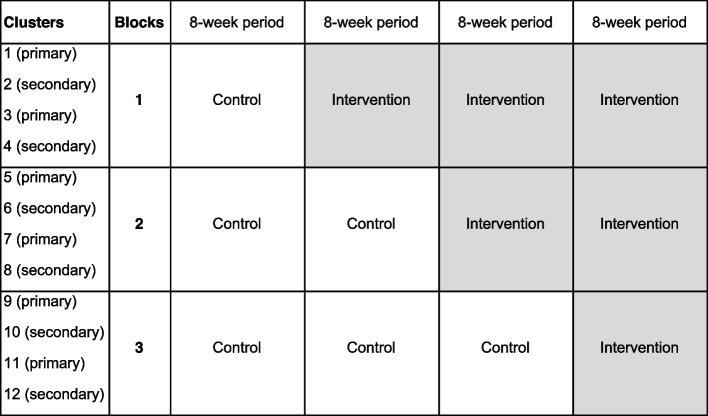
Stepped-wedge design for an evaluation of an arts-based intervention in Omoro, Uganda

The 32-week stepped-wedge cluster-randomized trial will be implemented in three blocks of four schools, each stratified by school type (primary, secondary), such that each block will consist of two schools of each type. The clusters are assembled into three blocks that will crossover from control to intervention condition, with crossover order selected at random. Including the control period, each school will participate across four 8-week periods with crossover from control to intervention condition occurring after each period [20, 21]. Clusters will receive the intervention over 24 weeks, 16 weeks, or 8 weeks depending on their block allocation (Fig. 2).

### Ethics and informed consent

This study has been approved by the Thompson Rivers University Research Ethics Board (102240) (Additional file 2), the University of Saskatchewan Behavioral Research Ethics Board (Beh-REB1701) (Additional file 3), and the University of Lethbridge Human Participant Research Committee (2020-004) (Additional file 4). In Uganda, the study has been approved by the Uganda National Council for Science and Technology (HS510ES) (Additional file 5) and the AIDS Support Organization (TASOREC/011/2020-UG-REC-009) (Additional file 6).

Information and consent forms (Additional file 7) will be provided to all participants, including students and their guardians/parents, teachers, and Elders. Forms will be drafted in English and translated to Luo. Verbal consent will be obtained prior to participation and recorded. Information and consent forms will inform participants that they may opt-out and withdraw their data at any time throughout the study; however, data cannot be withdrawn once the study has been completed. Participants will also be informed that all data will remain anonymous and confidential and that study results may be shared locally and internationally. This trial does not involve the collection of biological specimens.

### Conceptual framework

The adapted Health Stigma and Discrimination framework [22] will guide several aspects of this work, including the development and implementation of the arts-based intervention. The framework provides a theory of HIV stigma processes by highlighting various domains of health-related stigmatization across the socio-ecological spectrum. We have adapted the framework to the local context through a consultation process with the District Education Officer and the District Health Officer. Adaptation includes applying the framework’s constructs related to the drivers of stigma and facilitators of prevention to minimize the harmful effects of stigma during data collection and analysis.

### Study support and oversight

#### Community Advisory Committee

A Community Advisory Committee (CAC) will advise the research team on the development, implementation, refinement, and evaluation of the arts-based intervention and participate in the interpretation and dissemination of findings. The 16-member CAC is comprised of research team members and locals holding various leadership roles (e.g., Village Health Team, education specialists), community members, and youth in rural villages in Omoro District. Though intermittently disrupted by COVID-19 containment measures, community engagement by the research team began on 05/2019 and is ongoing. The CAC will be engaged for in-person and virtual meetings (or as per COVID-19 protocols) and will meet a minimum of twice per year. All meetings will be audio recorded with the consent of the participants.

#### Trial Steering Committee

The Trial Steering Committee (TSC) will consist of the study’s Principal Investigator (BF) and Co-Investigators (JM, GM, OB), a Research Associate (SCR), a representative of the CAC, and an external Academic Advisor. The TSC will approve the final study protocol, coordinate with study stakeholders and the Study Support Team, and oversee all aspects of the trial, including implementation of the intervention in school settings. The TSC will also review trial data, report study results, and contribute to knowledge translation and dissemination. The committee will meet once per month.

#### Study Support Team

The Study Support Team (SST) will consist of Project Coordinators and Research Assistants located in Omoro District, Uganda. The SST will execute tasks required for recruitment, training, data collection, and monitoring /evaluation activities.

### Setting and participants

This study will take place in Omoro District, Northern Uganda, where the HIV prevalence among the 15–24 age group at 7.2% is 20% greater than the national prevalence of 6.0% [23]. Omoro District has a slightly higher pregnancy rate among young people of 28% among 10–18 year-olds compared to the national rate of 25%. HIV prevalence among young pregnant mothers who were tested at their first antenatal visit was 8.9% in the district compared to 7.6% for pregnant mothers nationally [23].

In Omoro District, there are seven secondary and 65 primary government-funded schools that serve six sub-counties (Koro, Lalogi, Odek, Bobi, Ongako, Lakwana, and Omoro). Each grade level enrolls approximately 50 students per school, with approximately 12,000 students enrolled across these grades. In this study, we will approach students ≥10 years of age in six grade levels for recruitment. Primary grades 5–7 include approximately *n*=150 students per school; secondary grades 1–3 include *n*=150 students per school. Therefore, we estimate *n*=1800 as the total number of eligible students attending 12 schools across eligible grade levels.

### Randomization

In consultation with the District Education Officer, we identified a sampling frame of all government-funded schools situated within Omoro District (65 primary and seven secondary schools). One secondary school (Awere Secondary School) and nine primary schools (Awere, Dino, Odek, Agweno, Jing Komi, Acet, Awali, Aromo Wanglobo, and Binya) were considered ineligible as travel time from the research office in Omoro District to the school was estimated to be 1 h or longer. From the eligible sampling frame of 56 primary and six secondary schools, one Co-Investigator independently used a random number generator in Stata 11.0 to randomly select (without replacement) and allocate six primary and six secondary schools to three crossover blocks. Other Investigators, members of the local research team, and Head Teachers did not have advance knowledge of the allocation prior to enrollment. After allocation to blocks, Head Teachers were invited to enroll their schools in the study. Three primary schools (Ajuri, Laminonami, and Kweyo) were resampled due to a land issue that closed the school, a bad road that blocked transit to the school, and an administrative redistricting that moved the school to neighboring Gulu District. All secondary schools agreed to participate. The research team will coordinate with Head Teachers to facilitate participation of all classrooms in each eligible grade level.

### Recruitment of students

Initial contact with students will occur after schools are randomized and enrolled in the study. The research team will read the information letter in Luo in the classroom before inviting students to participate. A copy of the information letter will be sent home with students for parents or guardians to review with a choice to opt out. We will return within the following week to obtain a list of students who are interested in participating. Verbal consent (≥10 years old with parental opt-out process) will be sought and documented in a tracking form. Students who decline to participate may join another classroom during study activities.

We will recruit three classrooms per primary school and three per secondary school (one classroom per grade level with one participating teacher per classroom). Elders will also participate in the intervention with one Elder assigned per classroom. Each Elder will participate in the intervention in three different classrooms per week. An Elder, as defined by CAC members, is a person who is knowledgeable in traditional and customary issues, a custodian of cultural knowledge, and presides over many societal issues on behalf of their community. Elders will be recruited through consultation with each Head Teacher.

### Arts-based HIV stigma reduction intervention

#### Formative research phase

For pilot-testing, we will conduct six formative semi-structured interviews in Luo with one student and one teacher recruited by Head Teachers at one primary and secondary school, and two Elders. We will conduct an additional 50 one-on-one formative semi-structured interviews lasting 30–60 min, with purposively selected eligible students, Elders, and teachers at participating schools.

The initial interview guide will be created by the research team and revised with feedback from pilot interviews and the CAC to ensure cultural and contextual relevance. The interview guide will be drafted in English and translated to Luo. The guide will include questions for students, teachers, and Elders, such as “How are people who are known to be HIV-positive treated in your community, family, or school?” and “How does your village treat HIV-positive persons?” Data from the full set of formative qualitative interviews will be used to inform modifications to quantitative survey instruments.

In coordination with Head Teachers, 24 students aged ≥10 years, balanced by gender, and attending a participating primary or secondary school in grades primary 5–7 or secondary 1–3 will be invited to participate in one-on-one formative interviews. Additionally, 16 teachers will be recruited by the Head Teacher. Elders in the community will be identified based on competence in local language and demonstrated skills and practices in intergenerational knowledge transfer. Approximately five Elder women and five Elder men will be recruited through the Waroco Kwo Elders Association. The research team will attend an Elders’ meeting to facilitate discussions about the study and provide copies of the information letter/consent form. Interested Elders will contact the study’s research officer. Verbal informed consent will be obtained through information letters/consent forms translated and read to participants in Luo. Verbal consent from parents/guardians will be obtained for students. Participants will have the choice to opt out at any time.

#### Workshops

Following the formative research phase, one 3-day intervention training workshop will be delivered in collaboration with Elders, CAC members, teachers, and Head Teachers from participating schools. Workshops will serve to establish relationships between teachers and Elders, review findings of the formative phase, provide research ethics training, review the HIV-related stigma curriculum, and deliver several of the HIV stigma educational activities to practice facilitation of classroom discussions. The workshops will build the capacity for teachers and Elders to facilitate the intervention sessions with participating students in their classrooms.

#### Intervention components

The intervention will be delivered 2 h per week over 24 weeks and will consist of three interrelated activities: (1) transformative educational activities, (2) arts-based activities, and (3) participatory theater. These activities will address the drivers of HIV-related stigma (i.e., fear and misconceptions about HIV, lack of awareness, social judgment, shaming, prejudice, negative attitudes and reluctance to seek treatment or help), which manifest as types of stigma practices (i.e., enacted, internalized, anticipated, courtesy, perceived) [22].

Students will be eligible to participate in the arts-based intervention if they are ≥10 years of age, live in Omoro District in northern Uganda, attend a government-funded primary and/or secondary school in Omoro District during the study period, and are enrolled in primary grades 5–7 or secondary grades 1–3.

##### Transformative educational activities

Transformational educational activities will be based on Dewey’s [24] educational approach by grounding in the daily experiences of young people where stigmatizing experiences and practices are manifest. In the first hour of the weekly session, transformative educational activities will engage learners in problem-based discussions initiated with pictures from an HIV stigma toolkit that was created, implemented, and evaluated in sub-Saharan Africa with youth and adults [25]. The toolkit addresses drivers, facilitators, and manifestations of stigma as a means of articulating a “problem.” Learning through practice (i.e., participatory learning) will be emphasized through role play, discussion, and issue analysis.

##### Arts-based activities

These activities are anchored in aesthetics, i.e., theory of artful expression [26], and influenced by Eisner’s view that arts-informed research entertains, educates, and acknowledges individuals as knowledge-makers [27]. A key advantage of using art is to access “ways to perceive and interpret the world…that would otherwise go unknown” [27].

During the second hour of the weekly session, the focus will shift to addressing the “problem” through finding “solutions” and encouraging action by learning traditional Ugandan songs and dance; told and acted stories; proverbs and sayings that incorporate empowering messages and moral teachings connected with respect for the relationship between oneself and the community, peers and Elders; and responsibility for others. By grounding the intervention in local culture, this knowledge will reinforce the transformational learning needed to address the root causes of HIV stigma (e.g., stigma toward sex and adolescent sexual and reproductive health) while directly addressing each stigma type (i.e., enacted, internalized, anticipated, courtesy, and perceived). Drawing on the work of Paulo Freire, problem-based identification and discussion occurring in the first hour will be connected with development of problem-solving skills in the second hour [28]. Students will also be invited to write stories and music lyrics or create visual art that draws on their experiences with HIV-related stigma. The audio recordings of weekly sharing sessions will generate data for the process evaluation. Filming of storytelling sessions with Elders during the intervention will serve as knowledge preservation activities that promote intergenerational inclusion.

##### Participatory theater

After five weeks of intervention activies, a participatory theater intervention (PTI) will occur over the following 3-week period. Developed in collaboration with students, the PTI will summarize learning and cultural traditions from previous sessions. The first week will be dedicated to developing the forum theater event. The remaining two weeks will be dedicated to performances in front of audiences, including students, parents, and community members. Five topical scenes will be developed per school in collaboration with theater specialists, researchers, students, Elders, and teachers. Scenes will be developed through performance techniques [28] that will include a summary of qualitative data findings from formative interviews. Each scene will incorporate examples of stigma types (i.e., enacted, internalized, anticipated, courtesy, and perceived) with an HIV-positive and HIV-affected participant (student actor) who will perform each stigma domain. For example, student actors will portray a student who is living with HIV and experiencing internalized stigma and anticipated stigma; a student not living with HIV who is enacting stigma; a student who is experiencing courtesy stigma because of a mother living with HIV; and a student who perceives stigma in their community. Additionally, after enacted situations that present a problem with no solution, an audience member will be invited to enact a positive solution.

A semi-structured focused discussion will be held immediately after the PTI to explore participant and audience experiences. These discussions will be recorded and transcribed, providing qualitative data for the process evaluation.

### Process evaluation

Drawing on Linnan and Steckler’s framework [29], a process evaluation will be conducted throughout the intervention period. The process evaluation will be used to explain results and potential impacts on participants by assessing implementation per-protocol across study sites. This work will address four components of process evaluation: fidelity, sustainability, reach, and context [29]. Fidelity to the protocol will be achieved through standardized training and measurement of skill acquisition among Elders, teachers, and students throughout the intervention. Standardization of delivery will be supported by routine monitoring and review of the manualized intervention, observation of the classroom, and brief discussions with participants to assess facilitators and barriers to implementation. A protocol and manual outlining the process and content of the HIV stigma curriculum will be developed to support fidelity to intervention protocols [29].

Students, teachers, and Elder participants will be invited to participate in summative interviews to generate additional data for the process evaluation (5-10 students will be purposively selected from each of the three crossover groups). We will also randomly select ten CAC members to interview at the end of the study.

### Outcomes

Outcomes will include self-reported stigma-related measures and clinical measures. The primary outcomes are enacted, anticipated, and internalized stigma as measured using the *Adolescents Living with HIV—Stigma Scale* [30]. Secondary outcomes will include courtesy and perceived stigma measured using *Stewart et al.’s 10-item subscale* [31] and the *Brief Stigma by Association Scale* [32]. Secondary clinical outcomes will include HIV testing frequency among sexually active participants; and linkage to care, time to initiation of antiretroviral therapy (ART), ART adherence, and viral suppression, among participants living with HIV. Study outcomes will be assessed by independent research team members at baseline, following each crossover period (i.e., every 8 weeks), and 5-months after completion of the final intervention block.

### Power calculation

Using the power and detectable-difference calculations for stepped-wedge cluster-randomized trials (91) (Stata 14.2), we calculated 98% power to detect a 20% reduction (i.e., an absolute risk difference of 12%) in the proportion of young people reporting a stigma endpoint (i.e., endorsing sufficient elements in the primary stigma measure) given a baseline proportion of 60% experiencing HIV-related stigma, a 5% two-tailed type I error rate, and an intra-cluster correlation coefficient (ICC) of 0.01. There is a lack of published ICC estimates in this setting. Prior work in sub-Saharan Africa and rural Uganda found that 50% of participants experienced stigma in some form [33]. Given our team’s prior experience working among young people in areas recovering from conflict, we have assumed a higher baseline proportion of 60% to have experienced HIV-related stigma. We targeted a total cluster size of 12, with four schools (two primary and two secondary) grouped to crossover from control to intervention at three specified crossover points. We estimate that there will be 150 students enrolled per school across eligible grade levels. Assuming a 50% participation rate after attrition, we estimate that the evaluation will reach 75 students per school for a total sample of *n*=900 who will complete the surveys at the end of the final data collection period﻿ [34].

### Data collection and management

#### Survey design and measurements

A survey questionnaire will assess primary and secondary outcomes, along with selected covariates. The questionnaire will be developed in English and translated into Luo. The questionnaire will be refined with input from the CAC and pretested with 6–10 students to evaluate ease of comprehension, suitability of questions, and completion issues. Internal consistency of relevance scales (e.g., attitudes, stigma) will be assessed using Cronbach’s alpha.

The questionnaire will take up to 60 min to complete and will include questions about socio-demographics, reproductive and sexual health behavior, HIV testing, and HIV care. Stigma outcomes will be measured using three validated scales:

- Enacted, anticipated, and internalized stigma among HIV-positive students will be measured using the *Adolescents Living with HIV—Stigma Scale* [30].
- Courtesy stigma will be measured using the *Brief Stigma by Association Scale in HIV-affected students* [31].
- Perceived stigma will be measured using *Stewart’s 10-item subscale on felt-normative stigma towards PLHIV* in their community [32].

As intervention benefits may attenuate over time [35], we will examine sustainability of attitudinal change and stigma-associated change five months after the intervention has ended. Sexual risk, HIV testing frequency, and HIV status will be assessed by self-report.

#### Administrative health data

In partnership with TASO and community health clinics, health records will be used to assess clinical outcomes, including HIV status, date of confirmed diagnosis, time of ART initiation, viral suppression, and comorbid medical conditions. Data will be anonymized by sharing the unique Study ID file (linked to identifiers) with TASO, then receiving in return, data linked with the study ID stripped of all identifiers. These data will be linked by study ID within our de-identified dataset and remain confidential and anonymous. All data will be securely stored on a password-protected cloud-based platform, with access limited to approved members of the research team.

### Data analysis

#### Analysis of formative interviews

Interviews will be audio-recorded in Luo, transcribed, and translated in English. Transcription and translation will be conducted by local translators. Back-translation from English to Luo will be used to verify the accuracy of the original translation for 5–8 transcripts in order to enhance the trustworthiness of the findings [36, 37]. Final English transcripts and field notes will be imported into NVivo™ for analysis.

The Framework Method [38] will be used to analyze qualitative data. Researchers will begin by familiarizing themselves with the data by reading transcripts. The analytical process will begin with deductive coding guided by Stangl et al.’s [22] Health Stigma and Discrimination framework, followed by inductive coding for data that does not fit within the framework. Two researchers will independently code a subset of interviews, with a consensus code negotiated through peer-debriefing and coding comparisons undertaken in NVivo™. Agreed codes will be categorized to form an analytical framework which will be applied by researchers to code all subsequent interviews. Once all interviews are coded, data will be summarized using a matrix to facilitate interpretation and the generation of themes within the data [38]. Memos will be drafted throughout data analysis to record methodological decisions and analytical insights.

Descriptive and analytical strategies will be used to enhance rigor throughout qualitative data collection and analysis. The research team will maintain an audit trail of analytical steps and decisions taken throughout the research process. Peer-debriefing between researchers and members of the research team will enhance the trustworthiness and credibility of the findings. Member checking of findings [39] will occur within a focus group held with CAC members, which includes teachers and youth, to enable researchers to assess the validity of study findings [40]. CAC members will assist with the interpretation of findings in relation to participants’ narratives and validation of study conclusions.

#### Analysis of survey data

Descriptive statistics will be used to assess balance between clusters and to describe schools relative to the required sample size at each timepoint, socio-demographic characteristics, and primary/secondary outcomes. Main analyses will use linear mixed effect models (LMEM) or generalized linear mixed effect models (GLMEM) to assess the intervention effect. LMEM will be used for continuous outcomes that approach normality and GLMEM will be used for binary and other types of outcomes. Normality will be assessed using model residuals and outliers by Cook’s distance, leverage, and residuals. Time in weeks from the beginning of the trial, time in weeks centered at the beginning of the intervention, time in weeks from the beginning of school semesters, and blocks will be specified as fixed effects. The multilevel structure will be defined by assessments within participants as level one, participants as level two, and schools as level three. Schools and participants within schools will be specified as random effects. Main analyses will follow an intention-to-treat approach, with clusters analyzed as per the original treatment assignment. Sensitivity analyses will be performed to assess bias arising from attrition. A significance level of 0.05 will be used in all statistical tests. Analyses will be conducted in SAS v9.4.

### Data monitoring and adverse events

We have not established a Data Monitoring Committee as the arts-based intervention is considered to be low-risk. Similarly, stopping guidelines and interim analyses were not deemed necessary. Serious adverse events (SAEs) are not anticipated. Minor adverse events (AEs) may occur. For instance, children who live with HIV may be distressed while participating in discussions of stigma and discrimination. Additionally, it is possible that participants may report sexual assault that occurs within the community during the study timeframe, unrelated to study activities. In both cases, initial support will be offered through a trained counselor affiliated with the study. If further assessment or support is needed or the participant does not want to see the counselor, a list of referral agencies will be provided through our research team, who will support the participant to connect with the appropriate agency such as health, legal, livelihood, or security service providers. Any unanticipated SAE will be documented in relation to severity, expectedness, and causality, reported within seven calendar days to Ethics Committees, and discussed at a specially convened TSC meeting. AEs will be documented in the same fashion and discussed at the routine TSC meeting or sooner if an AE is repeated, and may be reported within 14 calendar days to Ethics Committees depending on severity and whether the event was anticipated. Furthermore, participants who report that they are sexually active and engage in condomless sex will be supported to attend the nearest health center for HIV testing and post-exposure prophylaxis as needed within 72 h, or will be provided with TASO’s contact information to receive appropriate counseling and support. Positive tests will be referred for counseling and follow-up per national protocols.

## Discussion

Addressing the stigma associated with HIV is critical for HIV prevention among young people, given associated harms in the form of adverse health and social outcomes, reduced access to HIV prevention services including HIV testing, and increased vulnerability to infection. Local cultural knowledge passed through Elders is an interventional approach that is culturally relevant and offers the potential for reducing HIV-related stigma among young people. Research in school settings has shown that the use of local cultural stories, songs, myths, riddles, and proverbs increases resilient coping responses among students [41] and strengthens positive and socially accepted morals and values [42]. Despite the importance of addressing HIV-related stigma among young people, a vulnerable population group that has experienced slower progress in HIV prevention than adults [43, 44], there is limited evidence supporting interventions that directly address stigma in school settings using local cultural knowledge to inform an art-based intervention.

Through a mixed-methods, community-based participatory research approach evaluated using a stepped-wedge cluster-randomized trial, we aim to generate in-depth, comprehensive data to examine the impact of an arts-based approach to HIV-related stigma intervention on key forms of stigma (i.e., enacted, internal, anticipated, courtesy, and perceived) and other HIV-related outcomes among HIV-positive and HIV-affected young people in Omoro District, Uganda. A process evaluation will characterize successes and challenges of the intervention in relation to effectiveness and suggest how to successfully implement arts-based  interventions in school settings, thereby increasing the transferability of our findings for global HIV policy and practice [29].

Due to stigma and discrimination around HIV, there may be a reluctance to participate in the intervention. Integration of the intervention within the context of a school setting as part of the curriculum may increase student participation. The stepped-wedge design will allow more students in the community to benefit from the intervention. However, implementing a new program may require additional effort on the part of teachers who have limited class time in which to incorporate the intervention curriculum. They may view learning a new curriculum as a difficult unpaid task within the context of their routine responsibilities. Training and program delivery for teachers will require a significant amount of additional time, commitment, and expertise. This may affect efficacy and fidelity, particularly in the context of added demands resulting from the COVID-19 pandemic. Schools in Uganda were closed from March 2020 to January 2022 (22 months) to reduce the spread of COVID-19. Vaccination rates in Uganda are low due to limited supply and significant vaccine hesitancy [45].

Given the longitudinal study design, there is the potential for participant attrition. COVID-19 will cause some students to stay home from school to avoid infection or to self-isolate upon the development of symptoms or a positive test. Young women participants of reproductive age may become pregnant during the study, which may cause them to drop out of school. Efforts will be made to retain this group by following up via text message and providing them with the option of continuing with intervention sessions without continuing with routine classroom attendance. We will contact students who are absent during follow-up data collection to minimize participant attrition. This intervention study has the potential to create a lasting impact within schools, surrounding communities, and to scientific understandings of ways to reduce stigma among young people.

### Trial status

The protocol version number is 2.0, dated July 29, 2022. Recruitment for formative interviews began on May 5, 2020. Study recruitment is expected to begin prior to the 2022–2023 school year. The target completion date is February 2024.

## Supplementary Information

**Additional file 1.** SPIRIT Checklist.

**Additional file 2.** Ethics Approval and Renewal - Thompson Rivers University. 

**Additional file 3.** Ethics Approval - University of Saskatchewan.

**Additional file 4.** Ethics Approval - University of Lethbridge.

**Additional file 5.** Research Approval - Uganda National Council for Science and Technology.

**Additional file 6.** Ethics Approval and Renewal - The AIDS Support Organization (TASO).

**Additional file 7.** Information Letters and Consent Forms.

**Additional file 8.** Funding Authorization - Canadian Institutes of Health Research.

## Data Availability

Datasets analyzed in this study, statistical codes, and the full study protocol are available from the corresponding author upon reasonable request. Materials will include audio-visual documentation of the arts-based intervention and Elders’ stories, which will be kept on a YouTube Channel that the Waroco Kwo Elders Association administers to promote intergenerational inclusion.

## Abbreviations

AEs: Adverse Events
ART: Antiretroviral therapy
CAC: Community Advisory Committee
COVID-19: Coronavirus Disease 2019
CBPR: Community-based participatory research
GLMEM: Generalized linear mixed effect models
ICC: Intra-cluster correlation coefficient
LMEM: Linear mixed effect models
PTI: Participatory theater intervention
SAEs: Serious Adverse Events
SST: Study Support Team
TASO: The AIDS Support Organization
TSC: Trial Steering Committee
