# Supplementary material for: Reducing HIV-related stigma among young people attending school in Northern Uganda: study protocol for a participatory arts-based population health intervention and stepped-wedge cluster-randomized trial
Source: Trials. 2022 Dec 23;23:1043. doi: 10.1186/s13063-022-06643-9 (PMC9782285; doi:10.1186/s13063-022-06643-9)
Supplement: Supplementary file 3 — Additional file 3. Ethics Approval - University of Saskatchewan. [file 13063_2022_6643_MOESM3_ESM.pdf]

UNIVERSITY OF SASKATCHEWAN

Behavioural Research Ethics Board (Beh-REB) 18-Dec-2020

Certificate of Approval

Application ID:	1701
Principal Investigator:	Bonnie Fournier<br>Geoffrey Maina
Locations Where Research Activities are Conducted:	Uganda
Student(s):	
Funder(s):	Canadian Institutes of Health Research
Sponsor:	Canadian Institutes of Health Research
Title:	Reducing HIV-related Stigma in School Children in Northern Uganda: A Multi-level Arts-based Population Health Intervention
Approved On:	18-Dec-2020
Expiry Date:	18-Dec-2021
Approval Of:	TRU Human Ethics Research Application<br><br>Information Letter for Verbal Consent - Baseline Interviews for Students<br><br>Information Letter for Verbal Consent - Arts-based Intervention with Youth 10-18 Years (Group 1, 2, and 3)<br><br>Information Letter for Verbal Consent - Arts-based Intervention for Teachers and Elders<br><br>Risk Management Plan during the COVID-19 Pandemic<br><br>Authorization to Release Confidential HIV Related Information: Parent/Guardian and Student<br><br>Baseline Questionnaire and Interview Questions
Acknowledgment Of:	
Review Type:	Full Board
Meeting Date:	12-Feb-2020

CERTIFICATION

The University of Saskatchewan Behavioural Research Ethics Board (Beh-REB) is constituted and operates in accordance with the current version of the Tri-Council Policy Statement: Ethical Conduct for Research Involving Humans (TPCS 2 2018). The University of Saskatchewan Behavioural Research Ethics Board has reviewed the above-named project. The proposal was found to be acceptable on ethical grounds. The principal investigator has the responsibility for any other administrative or regulatory approvals that may pertain to this project, and for ensuring that the authorized project is carried out according to the conditions outlined in the original protocol submitted for ethics review. This Certificate of Approval is valid for the above time period provided there is no change in experimental protocol or consent process or documents.

Any significant changes to your proposed method, or your consent and recruitment procedures should be reported to the Chair for Research Ethics Board consideration in advance of its implementation.

ONGOING REVIEW REQUIREMENTS

In order to receive annual renewal, a status report must be submitted to the REB Chair for Board consideration within one month prior to the current expiry date each year the project remains open, and upon project completion. Please refer to the following website for further instructions: https://vpresearch.usask.ca/researchers/forms.php.

Digitally Approved by Diane Martz
Chair, Behavioural Research Ethics Board
University of Saskatchewan
