# Supplementary material for: Reducing HIV-related stigma among young people attending school in Northern Uganda: study protocol for a participatory arts-based population health intervention and stepped-wedge cluster-randomized trial
Source: Trials. 2022 Dec 23;23:1043. doi: 10.1186/s13063-022-06643-9 (PMC9782285; doi:10.1186/s13063-022-06643-9)
Supplement: Supplementary file 4 — Additional file 4. Ethics Approval - University of Lethbridge. [file 13063_2022_6643_MOESM4_ESM.pdf]

University of Lethbridge

Office of Research Ethics
4401 University Drive
Lethbridge, Alberta, Canada
T1K 3M4
Phone: (403) 329-2747
Email: research.services@uleth.ca
FWA 00018802 IORG 0006429

Tuesday, 16 February 2021

PI:	Jean Harrowing, Faculty of Health Sciences
Study Title:	Reducing HIV-related Stigma in School Children in Northern Uganda: A Multi-level Arts-based Population Health Intervention
Action:	Approved
HPRC Protocol Number:	2020-004
Approval Date:	February 16, 2021
Term Date:	January 20, 2022

Dear Jean,

Thank you for submitting the renewal report for the study titled "Reducing HIV-related Stigma in School Children in Northern Uganda: A Multi-level Arts-based Population Health Intervention"; it has been reviewed and approved on behalf of the University of Lethbridge Human Participant Research Committee (HPRC) for the **approval period ending January 20, 2022**. The HPRC conducts its reviews in accord with University policy and the Tri-Council Policy Statement: Ethical Conduct for Research Involving Humans (2018).

Please note that an annual renewal report for continuing ethics certification will be due to the Office of Research Ethics on or before **January 20, 2022**.

We look forward to the resumption of this research when conditions allow.

Sincerely,

Susan Entz, M.Sc., Ethics Officer
Office of Research Ethics
University of Lethbridge
4401 University Drive
Lethbridge, Alberta, Canada
T1K 3M4
