# Supplementary material for: Reducing HIV-related stigma among young people attending school in Northern Uganda: study protocol for a participatory arts-based population health intervention and stepped-wedge cluster-randomized trial
Source: Trials. 2022 Dec 23;23:1043. doi: 10.1186/s13063-022-06643-9 (PMC9782285; doi:10.1186/s13063-022-06643-9)
Supplement: Supplementary file 5 — Additional file 5. Research Approval - Uganda National Council for Science and Technology. [file 13063_2022_6643_MOESM5_ESM.pdf]

Uganda National Council for Science and Technology
(Established by Act of Parliament of the Republic of Uganda)

Our Ref: HS510ES **17 November 2020**

Bonnie Fournier
TASO GULU UGANDA
Gulu

Re: Research Approval: **Reducing HIV-related Stigma in School Children**

I am pleased to inform you that on **17/11/2020**, the Uganda National Council for Science and Technology (UNCST) approved the above referenced research project. The Approval of the research project is for the period of **17/11/2020** to **17/11/2025**.

Your research registration number with the UNCST is **HS510ES**. Please, cite this number in all your future correspondences with UNCST in respect of the above research project. As the Principal Investigator of the research project, you are responsible for fulfilling the following requirements of approval:

1. Keeping all co-investigators informed of the status of the research.

2. Submitting all changes, amendments, and addenda to the research protocol or the consent form (where applicable) to the designated Research Ethics Committee (REC) or Lead Agency for re-review and approval **prior** to the activation of the changes. UNCST must be notified of the approved changes within five working days.

3. For clinical trials, all serious adverse events must be reported promptly to the designated local REC for review with copies to the National Drug Authority and a notification to the UNCST.

4. Unanticipated problems involving risks to research participants or other must be reported promptly to the UNCST. New information that becomes available which could change the risk/benefit ratio must be submitted promptly for UNCST notification after review by the REC.

5. Only approved study procedures are to be implemented. The UNCST may conduct impromptu audits of all study records.

6. An annual progress report and approval letter of continuation from the REC must be submitted electronically to UNCST. Failure to do so may result in termination of the research project.

Yours Sincerely

Hellen Opolot
For: Executive Secretary
UGANDA NATIONAL COUNCIL FOR SCIENCE AND TECHNOLOGY

LOCATION/CORRESPONDENCE

Plot 6 Kimera Road, Ntinda
P.O. Box 6884
KAMPALA, UGANDA

COMMUNICATION

TEL: (256) 414 705500
FAX: (256) 414-234579
EMAIL: info@uncst.go.ug
WEBSITE: http://www.uncst.go.ug
