# Supplementary material for: Reducing HIV-related stigma among young people attending school in Northern Uganda: study protocol for a participatory arts-based population health intervention and stepped-wedge cluster-randomized trial
Source: Trials. 2022 Dec 23;23:1043. doi: 10.1186/s13063-022-06643-9 (PMC9782285; doi:10.1186/s13063-022-06643-9)
Supplement: Supplementary file 7 — Additional file 7. Information Letters and Consent Forms. [file 13063_2022_6643_MOESM7_ESM.docx]

INFORMATION LETTER FOR VERBAL CONSENT (translated in Luo and read aloud) [Baseline Interviews with students]

# Study Title: Reducing HIV-related Stigma in School Children in Northern Uganda Who is conducting the study?

Dr. Bonnie Fournier, Thompson Rivers University Phone Number: + 1 250 318 7388 Dr. Olenka Bilash, University of Alberta

Dr. Geoffrey Maina, University of Saskatchewan Dr. Joshua Mendelsohn, Pace University

**Research Coordinator (Uganda**): Santo Ojok Phone Number: 0774 260 384

# Dear Student/Parent,

We want to tell you about a research study we are doing. We would like to find out more about HIV stigma and help deal with it in your school and community. You are being asked to join the study because HIV stigma is a problem for everyone including children who are HIV +. These children may be bullied or feared causing them to feel sad, alone, and to drop out of school. We would like to know more about how to prevent this from happening.

# What will you do in the study?

1. You will be asked 10-15 questions about HIV and your experience with HIV stigma during one visit. The visit will last 30-45 minutes in duration. The conversation/ talk will be recorded with a digital voice recorder and only heard by the research team.

# Why are you important to the study?

1. Your opinions and ideas are important “data” or information for the study.
2. We want to hear many different opinions – from boys, girls, teachers, and Elders. The more people we hear from, the better we can understand the situation.
3. The more we understand the better we can make suggestions for improving and eliminating HIV stigma in the future.

# Can anything bad happen?

If you are feeling sad or anxious and feel like you want to talk to someone, you can tell the research assistant, counselor or other staff or an adult you trust. Then we can help you by:

1. Making an assessment and providing any initial support to you.
2. If you would like to continue to see a counselor, please contact our project coordinator Santo Ojok for a resource referral sheet.
3. If you prefer not to see the counselor, the resource referral sheet will be provided to you by the research assistant.

# Who will know you are in this study?

1. Although no one will ever know your name, your words may be used in the results.
2. Any transcript of your interview will be stored in a very safe and secure place, namely on a password-protected device and encrypted or in a locked cabinet.
3. Only the research team in Canada or the research coordinator in Uganda will have access to these files.
4. They will be destroyed after five years following the end of the study. You will have the right to a report of the findings of this study at the end of the study by contacting the research coordinator Santo Ojok to receive a copy.

1

# How will others learn about the results of the study?

1. The results of the study will be shared with other professionals – teachers, nurses, doctors, health and education officials.
2. We will write articles about the results and will keep your name private, share them at conferences, local council meetings, or radio. Your name will not be attached to any result or included in any report unless you specifically request it.

# Do you have a choice?

1. You do not have to join this study. It is up to you. You can say okay now and change your mind later. All you have to do is tell us you want to stop during the visit. You do not need to answer any questions you do not want to. No one will be mad at you if you don’t answer questions, or don’t want to be in the study or if you join the study and change your mind later. If you decide to stop, all of your data will be destroyed and eliminated from the study. If you decide to stop, all of your data will be destroyed and eliminated from the study.
2. I understand that I cannot remove my information / data from the study after the study has been completed.
3. Before you say yes or no to being in this study, we will answer any questions you have. If you join the study, you can ask questions at any time by telling the research staff.

# Who do you contact if you have questions?

1. If you have any further questions regarding this study, please feel free to contact me - Bonnie Fournier at [bofournier@tru.ca](mailto:bofournier@tru.ca) or +1 250 318 7388 or the Research Coordinator Santo Ojok in Uganda at 0774 260 384.
2. For questions regarding participant rights and ethical conduct of research, contact the Research Ethics Office at [TRU-REB@tru.ca.](mailto:TRU-REB@tru.ca)

# Student Agreement to Participate

1. Yes, The research study has been explained to me.
2. Yes, I have been given the opportunity to ask questions and my questions have been answered.
3. Yes, I have been told whom to contact if I have more questions.
4. Yes, I have verbally agreed to participate in the research study described above and will receive a copy of this information letter.

# Parent/Guardian Agreement to Participate

1. Yes, The research study has been explained to me.
2. Yes, I have been given the opportunity to ask questions and my questions have been answered.
3. Yes, I have been told whom to contact if I have more questions.
4. Yes, I have verbally agreed to have my child in the research study described above and will receive a copy of this information letter.

2

INFORMATION LETTER FOR VERBAL CONSENT (translated in Luo and read aloud) [Arts-based Intervention with Youth >=10 years of age enrolled in primary grades 5-7 and secondary grades 1-3; Group 1, 2 & 3]

# Study Title: Reducing HIV-related Stigma in School Children in Northern Uganda Who is conducting the study?

Dr. Bonnie Fournier, Thompson Rivers University Phone Number: + 1 250 318 7388 Dr. Olenka Bilash, University of Alberta

Dr. Geoffrey Maina, University of Saskatchewan Dr. Joshua Mendelsohn, Pace University

**Research Coordinator (Uganda**): Santo Ojok Phone Number: 0774 260 384

# Dear Student,

We want to tell you about a research study we are doing in your school. A research study is a way to learn more about something. We would like to find out more about HIV stigma and help to deal with it in your school and community. Stigma refers to attitudes and beliefs that hurt other people in how they are treated. You are being asked to join the study because HIV stigma is a problem for everyone including children who are HIV-positive or children who have friends and relatives who are HIV-positive. These children may be bullied or feared causing them to feel sad, alone, and to drop out of school. We would like to know more about how to prevent this from happening.

# What will you do in the study?

- 1. Join a group of 50 students from your school.
  2. During your regular classroom period, discuss problems created by HIV and HIV stigma and solutions to make your school and community a safer place for everyone.
  3. Each week for 2 hours your teacher and two Elders will help you to discuss these problems and solutions using art (drawing, painting, singing, dancing).
  4. You will learn cultural stories, songs, dance and folklore each week as part of your learning about creating solutions.
  5. After 8 weeks, you will be asked if you would like to volunteer to join a theatre training for one week where you will have the opportunity to create 20 minute skits about HIV stigma.
  6. You will have the opportunity to perform those skits in front of other students and community members 5 times during a one week duration (Monday to Friday).
  7. You can participate in the art activities and decide not to participate in the theatre training or performance events.
  8. During specific time periods (beginning, end, and 5- months post study) you will be asked questions about how comfortable you feel like talking about HIV stigma to see if there have been any changes in your knowledge, attitude or behaviour.
  9. You will be assigned into one of three groups as part of the project. Each group will participate in the arts-based activities for either three semesters, two semesters or one semester.

# Why are you important to the study?

1. Your opinions and ideas are important provide important information.
2. We want to hear many different opinions – from boys, girls, teachers, and, Elders. The more people we hear from, the better can we understand how to make your school a safer place for everyone.
3. The more we understand the better can we make suggestions for improving and eliminating HIV stigma in the future.

# How will you benefit?

1. Gaining knowledge and skills regarding HIV and HIV stigma.
2. Practicing and improving your communication skills with other students and your teachers.
3. Learning more about your traditional cultural values and practices.
4. Helping other students and teachers benefit in the future from what we learn in the study.

# Could anything bad happen?

If, because of your participation in this project, you feel sad or anxious and feel like you want to talk to someone, you can tell the research assistant, counselor or other staff or an adult you trust. Then we can help you by:

1. Providing support to you.
2. Help to make an appointment for you with a counselor (our Project Coordinator, Santo Ojok will help you complete a referral sheet – please see top of this form for contact details).
3. If you prefer not to see the counselor, the resource referral sheet will be provided to you by the research assistant.

# Who will know you are in this study and how will the information you provide be used?

1. Other students in the study.
2. Although no one will ever know your name in our reports, if you allow us, your words may be used in the report we will write about the project.
3. Any arts-based work, transcripts, or digital audio of you will be stored safely and securely on a password-protected electronic device or in a locked cabinet.
4. Only the research team in Canada or the Project Coordinator in Uganda will have access to these files.
5. All research information will be destroyed after five years following the end of the study.
6. You will have the option to request a report of the completed findings by contacting the Project Coordinator (Santo Ojok) to receive a copy.
7. Some of your work such as your arts-based work (pictures, paintings, poems, songs, videos, and digital audios) may be analyzed in the future and used to write additional reports.
8. Your art work may be shown to others, but your name, your school and where you are from will never be revealed.

# Can you share things that other people say?

In agreeing to participate, you are agreeing to keep all that is said private. You will not share anything with students who are not in the study.

# How will others learn about the results of the study?

1. The results of the study will be shared with other professionals – teachers, nurses, doctors, health and education officials.
2. We will write articles about the results and will keep your name private, share them at conferences or even at local council meetings. Your name will not be attached to any result or included in any report.

# Do you have a choice?

1. You do not have to join this study. It is up to you. If you say “yes” now, you can change your mind later. All you have to do is tell us you want to stop. No one will be mad at you if you don’t want to be in the study or if you join the study and change your mind later. If you decide to stop, you will have the option of deleting all of the information you have provided to us. Deciding not to participate or to stop at any time will not affect your education or any other benefits you receive within or outside of school. If you decide to stop, all of your data will be destroyed and eliminated from the study.
2. I understand that I cannot remove my information / data from the study after the study has been completed.
3. Before you say yes or no to being in this study, we will answer any questions you have. If you join the study, you can ask questions at any time by telling the research staff.

# Who do you contact if you have questions?

If you have any further questions regarding this study, please feel free to contact me - Bonnie Fournier at [bofournier@tru.ca](mailto:bofournier@tru.ca) or +1 250 318 7388 or the Research Coordinator Santo Ojok in Uganda at 0774 260 384. For questions regarding participant rights and ethical conduct of research, contact the Research Ethics Office at [TRU-REB@tru.ca](mailto:TRU-REB@tru.ca)

# Agreement to Participate

- 1. Yes, The research study has been explained to me.
  2. Yes, I have been given the opportunity to ask questions and my questions have been answered.
  3. Yes, I have been told whom to contact if I have more questions.
  4. Yes, I have verbally agreed to participate in the research study described above and will receive a copy of this information letter.

INFORMATION LETTER FOR VERBAL CONSENT (translated in Luo and read aloud) [Arts-based Intervention for Teachers and Elders Participation in the Arts-Based Intervention]

# Study Title: Reducing HIV-related Stigma in School Children in Northern Uganda Who is conducting the study?

Dr. Bonnie Fournier, Thompson Rivers University Phone Number: + 1 250 318 7388 Dr. Olenka Bilash, University of Alberta

Dr. Geoffrey Maina, University of Saskatchewan Dr. Joshua Mendelsohn, Pace University

**Research Coordinator (Uganda**): Santo Ojok Phone Number: 0774 260 384

# Dear Teachers/Elders

We want to tell you about a research study we are doing in your school. A research study is a way to learn more about something. We would like to find out more about HIV stigma and help to deal with it in your school and community. Stigma refers to attitudes and beliefs that hurt other people in how they are treated. You are being asked to join the study because HIV stigma is a problem for everyone including children who are HIV-positive or children who have friends and relatives who are HIV-positive. These children may be bullied or feared causing them to feel sad, alone, and to drop out of school. We would like to know more about how to prevent this from happening.

# What will you do in the study?

1. You will be trained as facilitators to carry out the study in the classroom.
2. During the regular classroom period, you will discuss problems created by HIV and HIV stigma and solutions to make the school and community a safer place for everyone.
3. Each week for 2 hours you will help students in the classroom discuss these problems and solutions using art (drawing, painting, singing, dancing).
4. You will teach the students about their cultural stories, songs, dance and folklore each week as part of the students’ learning about creating solutions.
5. After 8 weeks, you will be asked if you would like to volunteer to join a theatre training for one week where you will have the opportunity to create 20 minute skits about HIV stigma with the students.
6. During specific time periods (beginning, end, and 5- months post study) you will be asked questions about how changes you are seeing in the students and your observations regarding the study.

# Why are you important to the study?

1. Your opinions and ideas are important provide important information.
2. You have expert knowledge in teaching and traditional knowledge that can help students learn how to deal with HIV stigma.
3. The more we understand the better can we make suggestions for improving and eliminating HIV stigma in the future.

# How will you benefit?

1. Gaining knowledge and skills regarding HIV and HIV stigma.
2. Practicing and improving your communication skills with other students and your teachers.
3. Learning more about your traditional cultural values and practices.
4. Helping students benefit in the future from what we learn in the study.

# Could anything bad happen?

If, because of your participation in this project, you feel sad or anxious and feel like you want to talk to someone, you can tell the research assistant, counselor or other staff or an adult you trust. Then we can help you by:

1. Providing support to you.
2. Help to make an appointment for you with a counselor (our Project Coordinator, Santo Ojok will help you complete a referral sheet – please see top of this form for contact details).
3. If you prefer not to see the counselor, the resource referral sheet will be provided to you by the research assistant.

# Who will know you are in this study and how will the information you provide be used?

1. Students, Elders and teachers in the study.
2. Although no one will ever know your name in our reports, if you allow us, your words may be used in the report we will write about the project.
3. Any arts-based work, transcripts, or digital audio of you will be stored safely and securely on a password-protected electronic device or in a locked cabinet.
4. Only the research team in Canada or the Project Coordinator (Santo Ojok) in Uganda will have access to these files.
5. All research information will be destroyed after five years following the end of the study.
6. You will have the option to request a report of the completed findings by contacting the Project Coordinator (Santo Ojok) to receive a copy.

# Can you share things that other people say?

In agreeing to participate, you are agreeing to keep all that is said private. You will not share anything with students, teachers or other Elders who are not in the study.

# How will others learn about the results of the study?

1. The results of the study will be shared with other professionals – teachers, nurses, doctors, health and education officials.
2. We will write articles about the results and will keep your name private, share them at conferences or even at local council meetings. Your name will not be attached to any result or included in any report.

# Do you have a choice?

1. You do not have to join this study. It is up to you. If you say “yes” now, you can change your mind later. All you have to do is tell us you want to stop. No one will be mad at you if you don’t want to be in the study or if you join the study and change your mind later. If you decide to stop, you will have the option of deleting all of the information you have provided to us. Deciding not to participate or to stop at any time will not affect your education or any other benefits you receive within or outside of school. If you decide to stop, all of your data will be destroyed and eliminated from the study.
2. I understand that I cannot remove my information / data from the study after the study has been completed.
3. Before you say yes or no to being in this study, we will answer any questions you have. If you join the study, you can ask questions at any time by telling the research staff.

# Who do you contact if you have questions?

If you have any further questions regarding this study, please feel free to contact me - Bonnie Fournier at [bofournier@tru.ca](mailto:bofournier@tru.ca) or +1 250 318 7388 or the Research Coordinator Santo Ojok in Uganda at 0774 260 384. For questions regarding participant rights and ethical conduct of research, contact the Research Ethics Office at [TRU-REB@tru.ca](mailto:TRU-REB@tru.ca)

# Agreement to Participate

- 1. Yes, The research study has been explained to me.
  2. Yes, I have been given the opportunity to ask questions and my questions have been answered.
  3. Yes, I have been told whom to contact if I have more questions.
  4. Yes, I have verbally agreed to participate in the research study described above and will receive a copy of this information letter.

INFORMATION LETTER FOR VERBAL CONSENT (translated in Luo and read aloud) [Workshops with teachers, Elders, and head teachers]

# Study Title: Reducing HIV-related Stigma in School Children in Northern Uganda Who is conducting the study?

Dr. Bonnie Fournier, Thompson Rivers University Phone Number: + 1 250 318 7388 Dr. Olenka Bilash, University of Alberta

Dr. Geoffrey Maina, University of Saskatchewan Dr. Joshua Mendelsohn, Pace University

**Research Coordinator (Uganda**): Santo Ojok Phone Number: 0774 260 384

# Dear Teacher/Elder/Headmaster,

We want to tell you about a research study we are doing in your school. A research study is a way to learn more about something. We would like to find out more about HIV stigma and help to deal with it in your school and community. Stigma refers to attitudes and beliefs that hurt other people in how they are treated. You are being asked to join the study because HIV stigma is a problem for everyone including children who are HIV-positive or children who have friends and relatives who are HIV-positive. These children may be bullied or feared causing them to feel sad, alone, and to drop out of school. We would like to know more about how to prevent this from happening.

# What will you do in the study?

1. You will be asked to join other teachers, Elders, and headmasters in a 4 day workshop.
2. You will learn more about HIV stigma and participate in some activities such as role plays to help you learn more during the first 2 days.
3. You will also learn more about the study intervention and your role and responsibility in the process in the final 2 days of the workshop.

# Why are you important to the study?

1. Your opinions and ideas are important “data” or information for the study.
2. We want to hear many different opinions – from teachers, Elders, and headmasters. The more people we hear from, the better we can understand the situation.
3. The more we understand the better we can make suggestions for improving and eliminating HIV stigma in the future.

# Can anything bad happen?

If you are feeling sad or anxious and feel like you want to talk to someone, you can tell the research assistant, counselor or other staff or an adult you trust. Then we can help you by:

1. Making an assessment and providing any initial support to you.
2. If you would like to continue to see a counselor, please contact our Project Coordinator Santo Ojok for a resource referral sheet.
3. If you prefer not to see the counselor, the resource referral sheet will be provided to you by the research assistant.

# Will I be compensated for participating in the study?

1. The cost of transportation to and from the workshops will be paid to you.
2. A meal will be provided during each workshop.

# Will I benefit from the study?

1. You will be helping your school and community be a safer place
2. You may increase your own knowledge of HIV stigma by participating in the interview

# Who will know you are in this study?

1. Although no one will ever know your name, your words may be used in the results.
2. Any transcript of your interview will be stored in a very safe and secure place, namely on a password-protected device and encrypted or in a locked cabinet.
3. Only the research team in Canada or the research coordinator in Uganda will have access to these files.
4. They will be destroyed after five years following the end of the study. You will have the right to a report of the findings of this study at the end of the study by contacting the research coordinator Santo Ojok to receive a copy.

# How will others learn about the results of the study?

1. The results of the study will be shared with other professionals – teachers, nurses, doctors, health and education officials.
2. We will write articles about the results and will keep your name private, share them at conferences or even at local council meetings.

# Do you have a choice?

1. You do not have to join this study. It is up to you. You can say okay now and change your mind later. All you have to do is tell us you want to stop during the workshop. You do not need to answer any questions you do not want to. No one will be mad at you if you don’t answer questions, or don’t want to be in the study or if you join the study and change your mind later. If you decide to stop, all of your data will be destroyed and eliminated from the study.
2. I understand that I cannot remove my information / data from the study after the study has been completed.
3. Before you say yes or no to being in this study, we will answer any questions you have. If you join the study, you can ask questions at any time by telling the research staff.

# Who do you contact if you have questions?

1. If you have any further questions regarding this study, please feel free to contact me - Bonnie Fournier at [bofournier@tru.ca](mailto:bofournier@tru.ca) or +1 250 318 7388 or the Research Coordinator Santo Ojok in Uganda at 0774 260 384.
2. For questions regarding participant rights and ethical conduct of research, contact the Research Ethics Office at [TRU-REB@tru.ca](mailto:TRU-REB@tru.ca)

# Agreement to Participate

1. Yes, The research study has been explained to me.
2. Yes, I have been given the opportunity to ask questions and my questions have been answered.
3. Yes, I have been told whom to contact if I have more questions.
4. Yes, I have verbally agreed to participate in the research study described above and will receive a copy of this information letter.

INFORMATION LETTER FOR VERBAL CONSENT (translated in Luo and read aloud) [Community Advisory Committee Membership (CAC)]

# Study Title: Reducing HIV-related Stigma in School Children in Northern Uganda Who is conducting the study?

Dr. Bonnie Fournier, Thompson Rivers University Phone Number: + 1 250 318 7388 Dr. Olenka Bilash, University of Alberta

Dr. Geoffrey Maina, University of Saskatchewan Dr. Joshua Mendelsohn, Pace University

**Research Coordinator (Uganda**): Santo Ojok Phone Number: 0774 260 384

# Dear Community Advisory Committee Member,

We want to tell you about a research study we are doing. We would like to find out more about HIV stigma and help it to disappear in your local schools and community. You are being asked to join the study to gain representation of community perceptions, preferences, and priorities in the development of our research project and research processes.

# What will you do in the study?

1. You will be asked to join a group of 20 people who will come together once a month (or more often as appropriate). These meeting will be audio recorded and transcribed to help guide and evaluate the study.
2. You will participate in four training workshops that will cover topics about HIV stigma and the arts-based intervention used in the study.
3. Serving in an advisory role you will provide information, guidance, or suggestions from your perspective regarding our research project and processes such as to:
   1. Review and approves all research materials we will use in the study.
   2. Review, guides and approves wording on all information sheets.
   3. Guide intervention development.
   4. Assist with evaluation of the project
   5. Assist with interpretation of the data that we will gather from the project
   6. Identify key issues for action and plan for the next steps in the research process.
4. You may be asked to talk with a member of the research team about your experiences and perceptions of being a member of the community advisory committee. The conversation/ talk will be recorded with a digital voice recorder and only heard by the research team.

# Why are you important to the study?

1. As a community member, your opinions and ideas are important to help guide our study.
2. We want to hear many different opinions –teachers, headmasters, individuals with HIV, boys, girls, local leaderships, and Elders. The more people we hear from, the better can we understand the situation.
3. The more we understand the better can we make suggestions for improving and eliminating HIV stigma in the future.

# Can anything bad happen?

1. The research team may choose a different decision than the community advisory committee’s decision. This could result in a misunderstanding between the research team and the CAC. to avoid this, the research team will make sure we listen and come to a consensus.
2. You may be inconvenienced by the scheduled meetings. We will make sure that everyone is in agreement with the time and day of each meeting.

# Will I be compensated for participating in the study?

1. The cost of transportation to and from the meetings will be paid to you.
2. A meal will be provided at each meeting.

# Will I benefit from the study?

1. Your participation in the research project will help us make sure that we understand the culture and the needs of the local community
2. Knowing that you are providing an important contribution to addressing HIV stigma in your community.

# Who will know you are in this study?

1. Although no one will ever know your name, your words may be used in the results.
2. Any transcript of your monthly meetings will be stored in a very safe and secure place, namely on a password-protected device and encrypted or in a locked cabinet.
3. Only the research team in Canada or the research coordinator in Uganda will have access to these files.
4. They will be destroyed after five years following the end of the study. You will have the right to a report of the findings of this study at the end of the study by contacting the research coordinator Santo Ojok to receive a copy.

# How will others learn about the results of the study?

1. The results of the study will be shared with other professionals – teachers, nurses, doctors, health and education officials.
2. We will write articles about the results and will keep your name private, share them at conferences or even at local council meetings.

# Do you have a choice?

1. You do not have to join this study. It is up to you. You can say okay now and change your mind later. All you have to do is tell us you want to stop. You can join the study and change your mind later. If you decide to stop, all of your data will be destroyed and eliminated from the study.
2. I understand that I cannot remove my information / data from the study after the study has been completed.
3. Before you say yes or no to being in this study, we will answer any questions you have. If you join the study, you can ask questions at any time by telling the research staff.

# Who do you contact if you have questions?

1. If you have any further questions regarding this study, please feel free to contact me - Bonnie Fournier at [bofournier@tru.ca](mailto:bofournier@tru.ca) or +1 250 318 7388 or the Research Coordinator Santo Ojok in Uganda at 0774 260 384.
2. For questions regarding participant rights and ethical conduct of research, contact the Research Ethics Office at [TRU-REB@tru.ca](mailto:TRU-REB@tru.ca)

# Agreement to Participate

1. Yes, The research study has been explained to me.
2. Yes, I have been given the opportunity to ask questions and my questions have been answered.
3. Yes, I have been told whom to contact if I have more questions.
4. Yes, I have verbally agreed to participate in the research study described above and will receive a copy of this information letter.

INFORMATION LETTER FOR VERBAL CONSENT (translated in Luo and read aloud) [Interviews with teachers, Elders]

# Study Title: Reducing HIV-related Stigma in School Children in Northern Uganda Who is conducting the study?

Dr. Bonnie Fournier, Thompson Rivers University Phone Number: + 1 250 318 7388 Dr. Olenka Bilash, University of Alberta

Dr. Geoffrey Maina, University of Saskatchewan Dr. Joshua Mendelsohn, Pace University

**Research Coordinator (Uganda**): Santo Ojok Phone Number: 0774 260 384

# Dear Teacher/Elder,

We want to tell you about a research study we are doing. We would like to find out more about HIV stigma and help deal with it in your school and community. You are being asked to join the study because HIV stigma is a problem for everyone including children who are HIV +. These children may be bullied or feared causing them to feel sad, alone, and to drop out of school. We would like to know more about how to prevent this from happening.

# What will you do in the study?

1. You will be asked 10-15 questions about HIV and your experience with HIV stigma during one visit. The visit will last 30-45 minutes in duration. The conversation/ talk will be recorded with a digital voice recorder and only heard by the research team.

# Why are you important to the study?

1. Your opinions and ideas are important “data” or information for the study.
2. We want to hear many different opinions – from boys, girls, teachers, and Elders. The more people we hear from, the better we can understand the situation.
3. The more we understand the better we can make suggestions for improving and eliminating HIV stigma in the future.

# Can anything bad happen?

If you are feeling sad or anxious and feel like you want to talk to someone, you can tell the research assistant, counselor or other staff or an adult you trust. Then we can help you by:

1. Making an assessment and providing any initial support to you.
2. If you would like to continue to see a counselor, please contact our project coordinator Santo Ojok for a resource referral sheet.
3. If you prefer not to see the counselor, the resource referral sheet will be provided to you by the research assistant.

# Who will know you are in this study?

1. Although no one will ever know your name, your words may be used in the results.
2. Any transcript of your interview will be stored in a very safe and secure place, namely on a password-protected device and encrypted or in a locked cabinet.
3. Only the research team in Canada or the research coordinator in Uganda will have access to these files.
4. They will be destroyed after five years following the end of the study. You will have the right to a report of the findings of this study at the end of the study by contacting the research coordinator Santo Ojok to receive a copy.

# How will others learn about the results of the study?

1. The results of the study will be shared with other professionals – teachers, nurses, doctors, health and education officials.
2. We will write articles about the results and will keep your name private, share them at conferences, at local council meetings or radio. Your name will not be attached to any result or included in any report unless you specifically request it.

# Do you have a choice?

1. You do not have to join this study. It is up to you. You can say okay now and change your mind later. All you have to do is tell us you want to stop during the visit. You do not need to answer any questions you do not want to. No one will be mad at you if you don’t answer questions, or don’t want to be in the study or if you join the study and change your mind later. If you decide to stop, all of your data will be destroyed and eliminated from the study. If you decide to stop, all of your data will be destroyed and eliminated from the study.
2. I understand that I cannot remove my information / data from the study after the study has been completed.
3. Before you say yes or no to being in this study, we will answer any questions you have. If you join the study, you can ask questions at any time by telling the research staff.

# Who do you contact if you have questions?

1. If you have any further questions regarding this study, please feel free to contact me - Bonnie Fournier at [bofournier@tru.ca](mailto:bofournier@tru.ca) or +1 250 318 7388 or the Research Coordinator Santo Ojok in Uganda at 0774 260 384.
2. For questions regarding participant rights and ethical conduct of research, contact the Research Ethics Office at [TRU-REB@tru.ca.](mailto:TRU-REB@tru.ca)

# Agreement to Participate

1. Yes, The research study has been explained to me.
2. Yes, I have been given the opportunity to ask questions and my questions have been answered.
3. Yes, I have been told whom to contact if I have more questions.
4. Yes, I have verbally agreed to participate in the research study described above and will receive a copy of this information letter.
