# Supplementary material for: Reducing HIV-related stigma among young people attending school in Northern Uganda: study protocol for a participatory arts-based population health intervention and stepped-wedge cluster-randomized trial
Source: Trials. 2022 Dec 23;23:1043. doi: 10.1186/s13063-022-06643-9 (PMC9782285; doi:10.1186/s13063-022-06643-9)
Supplement: Supplementary file 8 — Additional file 8. Funding Authorization - Canadian Institutes of Health Research. [file 13063_2022_6643_MOESM8_ESM.pdf]

Canadian Institutes of Health Research | Instituts de recherche en santé du Canada

AUTHORIZATION FOR FUNDING

CIHR (Canadian Institutes of Health Research) has approved funding as detailed below. Subject to the approbation of funding by Parliament, these funds will be made available to the business officer at the indicated institution for disbursement.

AUTORISATION DE FINANCEMENT

IRSC (Instituts de recherche en santé du Canada) vous accorde les fonds tel qu'indiqué ci-dessous. Suivant l'affectation des crédits par le Parlement du Canada, les fonds seront mis à la disposition du trésorier de l'établissement indiqué qui s'occupera des versements.

201809PJT-409282-PH1-ADZX-222914

04/02/2019

Institution Paid/Établissement chargé d'administrer les fonds:
Thompson Rivers University (Kamloops, BC)

Recipient(s)/Bénéficiaire(s): Fournier, Bonnie
School of Nursing
Thompson Rivers University (Kamloops, BC)

Dr. Joshua Mendelsohn, Mr. Santo Ojok, Dr. Geoffrey Muriithi Maina, Dr. Olenka Bilash

Program/Programme: Project Grant
Grant New

Primary Institute/Institut principal: Population and Public Health

Project Title/Titre du projet:

Reducing HIV-related Stigma in School Children in Northern Uganda: A Multi-level Arts-based Population Health Intervention

Co-investigator(s) & Associates/Supervisor(s)/Host/Co-chercheur(s)/Directeur(s) de recherche/Hôte:

Ms. Gillian Strudwick, Dr. Candace Nykiforuk, Dr. Kathryn Irene Banks, Dr. Jean N Harrowing, et al.

PAYMENT DETAILS/DÉTAILS DES VERSEMENTS — Funding Reference Number/ No. de Référence du financement: **PJT — 162310**

Period / Période	Type	Amount by Type / Montant par type	Total by Fiscal Year / Total par exercice	
01/04/2019 to 31/03/2020	Operating	$139,499	$139,499	2019-20
01/04/2020 to 31/03/2021	Operating	$139,499	$139,499	2020-21
01/04/2021 to 31/03/2022	Operating	$139,499	$139,499	2021-22
01/04/2022 to 31/03/2023	Operating	$139,499	$139,499	2022-23
01/04/2023 to 30/06/2023	Operating	$34,878	$34,878	2023-24

Progress Report Required: / Rapport des progrés réalisés requis:	**Not Applicable**	Application to Renew Funding Required: / Demande de renouvellement des fonds requis:	**Non-Renewable**

NOTES:

CIHR will require you to submit an electronic Final Report through the Research Reporting System on ResearchNet for this grant. Instructions will be provided through an email notification from the ResearchNet system once the activity becomes available.

CIHR requires that its contribution to your research be acknowledged in all written and oral presentations of your research results, including scientific articles, news releases, news conferences, public lectures and media interviews. For all scientific articles, the CIHR acknowledgement must include your Funding Reference Number (FRN) indicated above in the "Payment Details" section. Please see CIHR's Guidelines on Public Communication which are enclosed for more information on public communication and acknowledging requirements.

You received this funding because your colleagues volunteered their time to assist CIHR with the review of your application. We ask that, as a recipient of CIHR funding, you will participate in CIHR peer review activities if invited.

By drawing on the funds provided through this grant/award you agree to the terms and conditions set out in the attached "Conditions of Funding", any breach of which may result in CIHR taking remedial action as described therein.

If you are in receipt or become eligible to receive any funding from another source for any part of this project, you must advise CIHR immediately by following the instructions outlined in the "Funding Overlap Declaration" form http://www.cihr-irsc.gc.ca/e/797.html. Failure to self-declare overlap could lead to CIHR cancelling all funding related to this grant.

Renée Venne

Renée Venne
Acting Manager, Contact Centre
Operations Support

cc
___ Supervisor/Directeur
___ Dean/Doyen
___ Host/Hôte
___ Administration
___ Accountant/Comptable
___ CIHR Finance/Service des Finance d'IRSC
___ Other/Autre

Canada
