# Supplementary material for: Reducing HIV-related stigma among young people attending school in Northern Uganda: study protocol for a participatory arts-based population health intervention and stepped-wedge cluster-randomized trial
Source: Trials. 2022 Dec 23;23:1043. doi: 10.1186/s13063-022-06643-9 (PMC9782285; doi:10.1186/s13063-022-06643-9)
Supplement: Supplementary file 2 — Additional file 2. Ethics Approval and Renewal - Thompson Rivers University. [file 13063_2022_6643_MOESM2_ESM.pdf]

REB Approval (COA)

From: do-not-reply-TRU@researchservicesoffice.com [do-not-reply-TRU@researchservicesoffice.com]
Sent: Wednesday, December 11, 2019 1:15 PM
To: Bonnie Fournier
Cc: Truromeo; do-not-reply-TRU@researchservicesoffice.com
Subject: REB Approval (COA)

THOMPSON RIVERS UNIVERSITY

December 11, 2019

Dr. Bonnie Fournier
School of Nursing
Thompson Rivers University

File Number: 102240
Approval Date: December 11, 2019
Expiry Date: December 10, 2020

Dear Dr. Bonnie Fournier,

The Research Ethics Board has reviewed your application titled 'Reducing HIV-related Stigma in School Children in Northern Uganda: A Multi-level Arts-based Population Health Intervention'. Your application has been approved. You may begin the proposed research. This REB approval, dated December 11, 2019, is valid for one year less a day: December 10, 2020.

Throughout the duration of this REB approval, all requests for modifications, renewals and serious adverse event reports are submitted via the Research Portal. To continue your proposed research beyond December 10, 2020, you must submit a Renewal Form before December 10, 2020. If your research ends before December 10, 2020, please submit a Final Report Form to close out REB approval monitoring efforts.

If you have any questions about the REB review & approval process, please contact the Research Ethics Office via 250.852.7122. If you encounter any issues when working in the Research Portal, please contact the Research Office at 250.371.5586.

Sincerely, Joyce O'Mahony
Chair, Research Ethics Board

Subject: REB Renewal/Amendment Approval
Date: Tuesday, November 10, 2020 at 2:24:30 PM Eastern Standard Time
From: do-not-reply-TRU@researchservicesoffice.com
To: Bonnie Fournier
CC: Truromeo, do-not-reply-TRU@researchservicesoffice.com

THOMPSON RIVERS UNIVERSITY

November 10, 2020

Dr. Bonnie Fournier
School of Nursing
Thompson Rivers University

File Number: 102240
Original Approval Date: December 10, 2019
Most Recent Renewal: November 10, 2020
Expiry Date: December 10, 2021

Dear Dr. Bonnie Fournier,

The Research Ethics Board has reviewed your renewal/amendment for the project titled 'Reducing HIV-related Stigma in School Children in Northern Uganda: A Multi-level Arts-based Population Health Intervention'. Your renewal/amendment has been approved. You may begin the proposed research. This REB approval, dated December 10, 2019, is valid for one year: December 10, 2021.

Throughout the duration of this REB approval, all requests for modifications, renewals and serious adverse event reports are submitted via the Research Portal. To continue your proposed research beyond December 10, 2021, you must submit a Renewal Form before December 10, 2021. If your research ends before December 10, 2021, please submit a Final Report Form to close out REB approval monitoring efforts.

If you have any questions about the REB review & approval process,please contact the Research Ethics Office via 250.852.7122. If you encounter any issues when working in the Research Portal, please contact the Research Office at 250.371.5586.

Sincerely,

Joyce O'Mahony
Chair, Research Ethics Board
