# Supplementary material for: Reducing HIV-related stigma among young people attending school in Northern Uganda: study protocol for a participatory arts-based population health intervention and stepped-wedge cluster-randomized trial
Source: Trials. 2022 Dec 23;23:1043. doi: 10.1186/s13063-022-06643-9 (PMC9782285; doi:10.1186/s13063-022-06643-9)
Supplement: Supplementary file 6 — Additional file 6. Ethics Approval and Renewal - The AIDS Support Organization (TASO). [file 13063_2022_6643_MOESM6_ESM.pdf]

Approval Notification for Initial Review Application: Protocol # TASOREC/011 /2020-UG-REC-009

Sent: Monday, April 20, 2020 2:53 AM
Subject: Approval Notification for Initial Review Application: Protocol # TASOREC/011/2020-UG-REC-009

Dear Prof. Bonnie Fournier,

TASO IRB/REC reviewed your correspondence with revisions to the non-academic research protocol,

Protocol REC Application Title**:** **Reducing HIV-Related Stigma in School Children in Northern Uganda: A Multi-level Arts-Based Population Health Intervention**

In the matter concerning the review, we are pleased to confirm a favorable ethical decision for the above research project.

Effective **17/04/2020** the REC approved the initial application request for the research.
Protocol expiration date: **16/04/2021**
Annual review due date: **15/02/2021**

To Note

Submit two final clean versions (hard copies) of the revised protocol bearing the date and version number, data collection instrument (s), and consent/assent forms with translations (where applicable) for approval with a TASO REC Stamp.

You will also be availed with the ethics approval letter upon submission of the foregoing.

The approval provides permission to begin the research activities outlined in the REC-approved protocol and supporting documents.

All research activities must be conducted in accordance with only approved (stamped) protocols.

We wish you success in the conduct of your study.

Please contact TASO REC if you have any questions or need additional information.

Sincerely,

Mijumbi Andrew Ojok,
TASO IRB/REC Administrator
+256 785037496; 752774281

The AIDS Support Organisation (TASO) Uganda Ltd.

TASO Headquarters
Mulago Hospital Complex
P.O. Box 10443, Kampala-Uganda
Tel: +256 414 532 580/1
Fax: +256 414 541 288
Email: mail@tasouganda.org
Website: www.tasouganda.org

TASO COLLEGE OF HEALTH SCIENCES (TACHS)
Kanyanya Off Gayaza-Road,
After Mpererwe
P.O. Box 10443, Kampala
Tel: +256 414 567 637
Fax: +256 414 566 704
Email: training@tasouganda.org

SERVICE CENTRES

TASO ENTEBBE
Plot 15-17 Lugard Avenue
P.O. Box 235, Entebbe
Tel: 0414 320 030/0752 774 135
Email: entebbe@tasouganda.org

TASO GULU
Plot 4 Mathew Lukwiya Road
P.O. Box 347, Gulu
Tel: 0471 432743/ 0752 774142
Email: gulu@tasouganda.org

TASO JINJA
Jinja Referral Hospital
P.O. Box 577, Jinja
Tel: 0393260 117/0752 774 145
Fax: 0434 120382
Email: jinja@tasouganda.org

TASO MASAKA
Masaka Hospital
P.O. Box 1679, Masaka
Tel: 0392 749 998/0752 774 145
Email: masaka@tasouganda.org

TASO MASINDI
Masindi Hospital
P.O. Box 117, Masindi
Tel: 0465 420 636/ 0752 774 144
Fax: 0465 420 636
Email: masindi@tasouganda.org

TASO MBALE
Mbale Hospital
P.O. Box 2250, Mbale
Tel: 0454 433 507/ 0752 774 137
Fax: 0454 435 851
Email: entebbe@tasouganda.org

TASO MBARARA
Plot 25 and 27 Hospital Road
P.O. Box 1010, Mbarara
Tel: 0485 421 323/ 0752 774 136
Fax: 0485 421 323
Email: mbarara@tasouganda.org

TASO MULAGO
Mulago Hospital
P.O. Box 11485, Kampala
Tel: 0414 530 034/ 0752 774 139
Fax: 0414 541 288
Email: mulago@tasouganda.org

TASO RUKUNGIRI
Ishasha Road
P.O. Box 350, Rukungiri
Tel: 0486 442 610/ 0752 774 141
Fax: 0486 442 613
Email: rukungiri@tasouganda.org

TASO SOROTI
Soroti Hospital
P.O. Box 422, Soroti
Tel: 0454 461 380/0752 774 143
Fax: 0454 461 042
Email: soroti@tasouganda.org

TASO TORORO
Plot 30, Cox Road
P.O. Box 777, Tororo
Tel: 0454 442 009/0752 774 140
Fax: 0454 445 334
Email: tororo@tasouganda.org

DISCRETE PROJECTS

GRANTS MANAGEMENT UNIT / GLOBAL FUND
House of Hope, Plot 10
Windor Loop
P.O. Box 10443, Kampala
Tel: 0414 259 555/ 0752 774 109
Email: mail@tasouganda.org

TASO-KARAMOJA PROJECT
Plot 10, Independence Avenue
P.O. Box 131, Moroto
Tel: 0752 744 792
Fax: 0454 445 334
Email: mail@tasouganda.org

TORORO LABORATORY HUB
P.O. Box 777, Tororo
Tel: 0454 442 009/ 0752 774 723
Fax: 0454 445 334

16th April, 2021

Our Ref: TASOREC/011/2020-UG-REC-009 (RNWL)

Dr. Bonnie Fournier,
Principal Investigator
bofournier@tru.ca

Dear Dr. Bonnie,

RE: ANNUAL RENEWAL OF RESEARCH PROTOCOL **"REDUCING HIV-RELATED STIGMA IN SCHOOL CHILDREN IN NORTHERN UGANDA: A MULTI-LEVEL ARTS-BASED POPULATION HEALTH INTERVENTION.**"

Thank you for submitting the progress report and an application for annual renewal for the protocol titled **"Reducing HIV-Related Stigma in School Children in Northern Uganda: A Multi-level Arts-Based Population Health Intervention"** Version 2 dated, **23rd March 2021.**

In the matter concerning the continuing review of the application and progress report, the committee has renewed the study for one year.

This approval is valid until **15th April 2022,** after which you will be required to make a request for extension to the Chairperson TASO REC in case of continuation with the study.

Study Reports: It is a requirement by the REC that you submit timely progress reports.

Proceed with the registration of the continuing review approval by the Uganda National Council of Science and Technology (UNCST) before commencement.

Yours sincerely,

THE AIDS SUPPORT ORGANISATION
15 APR 2022
Approved, Valid Until..........
RESEARCH ETHICS COMMITTEE

Dr. Jjuuko Adrian,
Chairperson, TASO RESEARCH ETHICS COMMITTEE (REC)

CC: Executive Director, TASO (U) Limited
CC: Uganda National Council for Science & Technology (UNCST)
